# Case report: Persistent COVID-19 in a patient with B cell lymphoma refractory to antiviral treatment due to resistance to Remdesivir

**DOI:** 10.1016/j.idcr.2025.e02199

**Published:** 2025-03-22

**Authors:** Luiz Eduardo Ceccon Calil de Assumpção, Bruno Graciano Ponce Romeo, João Carlos de Campos Guerra, Luis Fernando Aranha Camargo, Marcelo Akira Nagaoka, Deyvid Emanuel Amgarten, Erick Gustavo Dorlass, Roberta Cardoso Petroni, Afonso Celso Almeida Cardoso, Renato de Mello Ruiz, Carolina Devite Bittante, Vanessa Damazio Teich, João Renato Rebello Pinho, André Mario Doi

**Affiliations:** a School of Medicine, Faculdade Israelita de Ciências da Saúde Albert Einstein, São Paulo, Brazil; b Hospital Israelita Albert Einstein, Brazil

**Keywords:** COVID, Mutation, Resistance, SARS-CoV-2, Remdesivir, Antiviral, Immunosuppressed, Coronavirus

## Abstract

***Background*:**

There is a significant concern of the pandemic impact of SARS-CoV-2 infection in immunocompromised patients. These patients can develop long COVID-19 due to impairment of cellular and humoral immunity. On the other hand, prolonged infection can lead to mutations in the SARS CoV-2 genome that can impact on the resistance to antiviral therapy. Remdesivir cases have been reported in patients receiving antiviral drug treatment.

**Case presentation:**

A 46-year-old male with previous mantle cell lymphoma resolved by autologous bone marrow transplantation without other comorbidities had SARS-CoV-2 detected in February 2022 and received the recommended antiviral treatment with Remdesivir. COVID-19 evolved in four months with worsening of the symptoms, despite an initial rapid improvements and high RT-PCR Ct values. The patient was relieved from hospital care stable and well but still maintaining positive test results.

**Conclusions:**

the patient presented prolonged COVID-19 with persistence of virus detected by RT-PCR for several months. The strain sequenced presented a mutation different from all reported previously. Although it was no possible to sequence the initial strain without these mutations, our data suggests that immunocompromised patient with prolonged COVID-19 may serve as reservoir for strains of SARS-CoV-2 with resistant components in his genome.

## Background

There is significant concern about the impact of SARS-CoV-2 infection in immunocompromised patients, as these individuals may develop long COVID due to impaired immune responses. Furthermore, prolonged infections in these patients can result in mutations in the SARS-CoV-2 genome, potentially reducing the effectiveness of antiviral treatments. Cases of Remdesivir resistance in patients with underlying immunosuppression have already been reported in the medical literature, supporting the statement this article intends to make [1], [2], [3], [4].

### Case summary

A 46-year-old male with a history of mantle cell lymphoma (resolved with autologous bone marrow transplantation) and no other comorbidities, was evaluated positive for SARS-CoV-2 in February 2022. Although the patient initially received the recommended antiviral treatment with Remdesivir, showing rapid improvement and high RT-PCR CT values, his symptoms and laboratory markers worsened over the next four months. The patient was, eventually, discharged from the hospital in stable clinical status, although he continued to be positive for SARS-CoV-2 (Fig. 3).

#### Conclusions: a summary of the clinical impact or potential implications of the case report

We concluded in this report that even going through the recommended anti-viral treatment, the patient presented prolonged covid-19 with persistence of virus detected by RT-PCR for more than four months. The sequenced viral strain presented a mutation described only in “in vitro” studies and cases reported with no clinical data. Although it was not possible to sequence the initial viral-strain without these mutations, which shows the main flaw of our report, the data suggests that an immunocompromised patient with prolonged covid-19 may serve as reservoir for the development of new strains of SARS CoV-2 with resistant components in its genome.

## Case report

A 46-year-old patient previously diagnosed with mantle cell lymphoma, submitted to an autologous stem cell transplantation in October 2020, was admitted to hospital 02/01/2022 with fever, myalgia, tiredness, and oxygen saturation of 97 %. The patient had been on maintenance doses of rituximab for 1 year and 4 months.

The antigen test for *Influenza* A/B was negative, and SARS-CoV-2 antigen tested positive. A thorax computerized tomography (CT) demonstrated multiple ground-glass opacities and consolidations with bilateral multifocal distribution and greater involvement of the inferior lobes (less than 50 % of involvement). The patient was hospitalized and inserted in a ten-day treatment regimen of Remdesivir, Moxifloxacin and corticosteroids. Five days later, he was discharged, stable, with improved symptoms.

However, ten days after discharge, the patient was readmitted with worsening symptoms. The thorax CT pattern also showed an extension of ground-glass opacities and new areas of consolidation. Once he had already used Remdesivir, it was prescribed methylprednisolone and cefepime. During the hospitalization, from 02/15/2022-02/25/2022, the patient stayed without fever and kept stable respiratory parameters despite a control CT showing an increase of ground-glass opacities and atelectasis. On 03/01/2022, he returned with fever, prostration, and desaturation (95 %).

During this stay, *Bordetella bronquipseptica* had grown on bronchoalveolar lavage (BAL), so clarithromycin and meropenem were prescribed. The patient evolved with pneumomediastinum post non-invasive ventilation (NIV). The patient also received immunoglobulin. A transbronchial biopsy showed fibrous thickening of alveolar septa and intra-alveolar fibrosis. On 03/14/2022, the patient presented hypoxemia and worsening of ventilatory parameters, needing a new course of Remdesivir for seven days and transferring to the intensive care unit. In the ICU, Paxlovid was also introduced for 6 more days from 03/22/2022-03/28/2022. The patient remained stable with an improvement of respiratory patterns but evolved with infection by *Cytomegalovirus* (548 UI/mL log 2.74) due to immunosuppression and pneumonia by *Klebsiella pneumoniae* and *Pseudomonas aeruginosa*. He was treated with meropenem and ganciclovir.

The patient remained hospitalized until May 15, 2022, with an improvement in clinical condition despite respiratory sequelae, as evidenced by abnormalities in respiratory function tests and serial CT scans showing diffuse pulmonary infiltration and ectasia of the bronchi and bronchioles. The patient was discharged on catheter oxygen therapy and continued to test positive for SARS-CoV-2 by RT-PCR.

During clinical follow-up, serial RT-PCR tests for SARS-CoV-2 were conducted, revealing persistent detection of viral RNA in samples for nearly four months. Fig. 2 illustrates the serial RT-PCR results over time along with the corresponding Ct (cycle threshold) values. After this prolonged period, the patient showed improvement in both clinical and tomographic status, with subsequent negative RT-PCR results for SARS-CoV-2.

At present, the patient remains clinically stable and no longer requires oxygen therapy.

## Results

Throughout the course of treatment, RT-PCR cycle threshold (Ct) values remained relatively high, indicating a significant viral load even after Remdesivir was introduced. This suggests that viral persistence contributed to prolonged pulmonary injury in the patient. Nasopharyngeal and throat swab samples with Ct values below 30 were processed for whole genome sequencing to identify the SARS-CoV-2 lineage and examine any mutations.

**Fig. 1:**
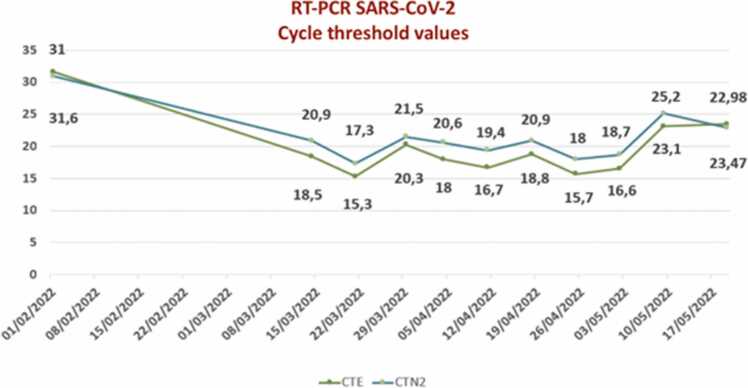
RT-PCR SARS CoV-2 showing Ct levels since first diagnosis.

RNA was extracted using the MagNA Pure 96 System (Roche), and whole genome sequencing was performed using the COVIDSeq protocol by Illumina with the NextSeq 550 platform. The resulting sequences were identified as the BA.1 (Omicron) lineage (Fig. 1 – A) using Pangolin software (v4.0.6). Further sequencing revealed an E796D SNP mutation in the RdRP protein (ORF1ab – nsp12), which altered its structural conformation by creating a three-helix GGG residue compared to the original BA.1 strain (Fig. 1 – B). This mutation is located near the previously reported E802D mutation, which is known to cause Remdesivir resistance. While the E796D mutation has not been directly linked to antiviral resistance, the E796G mutation has been shown to confer some resistance to Remdesivir in vitro (Fig. 4).

**Fig. 2:**
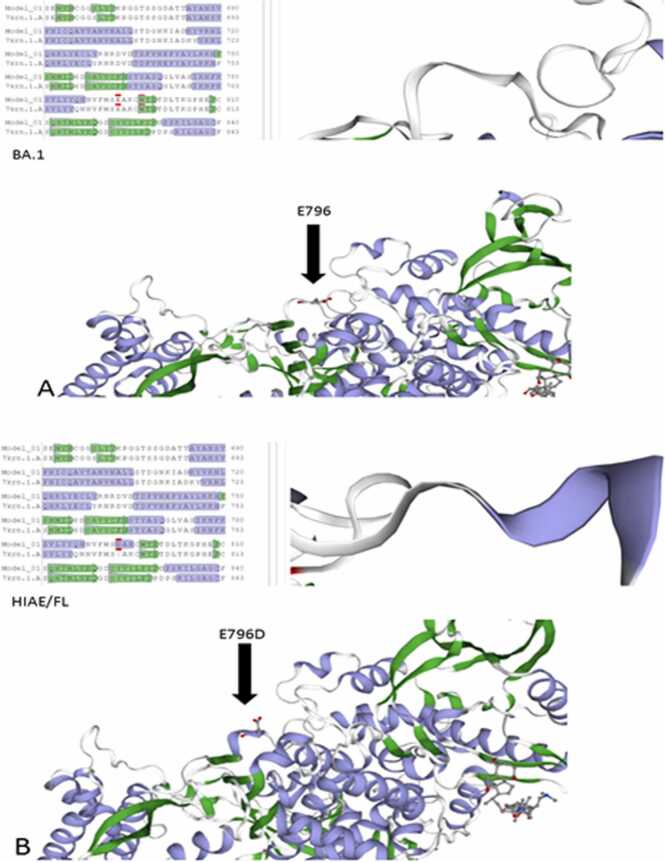
**A** RdRP modeling from sample BA.1 (omicron). Arrow points to the sequence in study.*Fig. 2**B** RdRP modeling from sample HIAE/FL. Arrow points SNP position and subsequent 3 – helix GGG structural alteration.*. *Fig. 2 A and B were obtained via SWISS Prot Software [5].

**Fig. 3:**
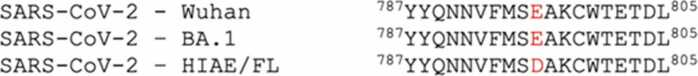
Main RdRP sequence comparison between the original Pandemic variant, omicron and the one presented in this article.

**Fig. 4:**
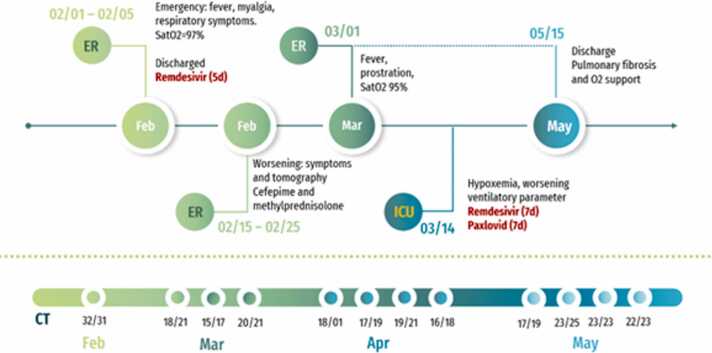
Infographic resuming main events in FLF’s clinical history. 1 Contributed equally to this work.

Raw sequences were directly mapped to the SARS-CoV-2 reference genome (Genbank NC_045512.2) with BWA v0.7.17 [6]. The mapping metrics are collected with Samtools v1.7.0 [7]. Variants are called with HaplotypeCaller v4.0.5.1 [8]. Gene annotations were performed with annovar software version 2020jun08 [9]. The consensus sequence was generated with Bcftools v1.9, and finally lineage classification was carried out with Pangolin v4.0.6. [7].

## Discussion and conclusion

We report a case of prolonged COVID-19 infection in an immunosuppressed patient treated with remdesivir and Paxlovid, with limited clinical response to these antivirals. This case highlights that immunosuppression—and the resulting inability of the immune system to effectively clear the virus—may not only facilitate prolonged COVID-19 infections but also potentially contribute to the development of spike protein mutations in viral strains, leading to treatment resistance and worse clinical outcomes. The patient was using rituximab for 1 year and 4 months and clinically immunosuppressed with a current CMV infection with high viral load.

Although the initial naïve viral strain prior to treatment was not recovered, our data support the hypothesis that prolonged infections, particularly in patients receiving immunosuppressive treatments, like Rituximab, may promote the emergence of mutations that contribute to antiviral resistance and poorer clinical outcomes, as already reported in current literature [10], [11], [12], [13], [14], [15], [16].

The E796D mutation in the gene encoding the RNA-dependent RNA polymerase (RdRp), specifically in the non-structural protein 12 (NSP12) of SARS-CoV-2, is essential for viral replication. Recent studies have linked this mutation to resistance, demonstrating RDV-resistant mutations through in vitro passages of SARS-CoV-2 in the presence of RDV. [15] Additionally, Torii et al. showed that the infection rates of presumed RDV-resistant mutant viruses were higher or significantly increased [14], [16].

Clinical samples collected in Manaus, Amazonas, Brazil, between 2020 and 2022 revealed the same mutations, although no clinical data were detailed. The publication shows that non-structural protein 12 sequences from SARS-COV-2 found in Manaus, Amazonas, Brazil, reveals mutations linked to higher transmissibility. This highlights the concern and the importance of monitoring these mutations and variants resistant to new antivirals [17].

Furthermore, Rituximab treatment has also been identified as a risk factor for long-term SARS-CoV-2 persistence and associated with severe disease and death. Our patient had been on maintenance doses of rituximab for 1 year and 4 months corroborating with this evidence [18].

Therefore, our findings, along with the literature review, highlight the importance of closely monitoring viral mutations in immunosuppressed patients, especially those who fail to respond to standard antiviral therapies. We have shown that the E796D mutation reported in this case may serve as an important marker for potential resistance to Remdesivir, however, further research is still needed to better understand its role in clinical outcomes. As new mutations emerge, it will be crucial to adapt our treatment strategies to ensure the best possible outcomes for these vulnerable patients.

## List of abbreviations

FDA

SNP (single nucleotide polymorphism)

SRS-CoV-2

RT-PCR

CT (computerized tomography)

RNA

TLC (total lung capacity)

COVID-19

## Ethics approval and consent to participate

The present study states that all the ethics approval and consent were performed.

## Author agreement

All authors have seen and approved the final version of the manuscript being submitted.

## Consent for publication

Podem pedir uma cópia em qualquer memento.

## Author statement

LECCA, BGPR, JRRP, and AMD have made substantial contributions to the conception, design, acquisition and analysis of the data; JCCG, LFAC, MAN, DEA, EGD, RCP, and ACAC have made substantial contributions to the conception, design, draft of the work and revision of the work; RMR, CDB, and VDT have made substantial contributions to the design and revision of the work. All authors read and approved of the final manuscript.

## CRediT authorship contribution statement

**Pinho João Renato Rebello:** Writing – review & editing, Conceptualization. **Romeo Bruno Graciano Ponce:** Writing – original draft, Formal analysis. **Teich Vanessa Damazio:** Writing – review & editing, Formal analysis. **Amgarten Deyvid Emanuel:** Writing – review & editing, Data curation. **Nagaoka Marcelo Akira:** Writing – review & editing, Validation. **Camargo Luis Fernando Aranha:** Writing – review & editing, Conceptualization. **Doi André Mario:** Writing – review & editing, Writing – original draft, Formal analysis, Conceptualization. **Guerra João Carlos de Campos:** Writing – review & editing, Conceptualization. **Ruiz Renato de Mello:** Writing – review & editing, Data curation. **Cardoso Afonso Celso Almeida:** Writing – review & editing, Validation. **Petroni Roberta Cardoso:** Writing – review & editing, Validation. **Dorlass Erick Gustavo:** Writing – review & editing, Conceptualization. **de Assumpção Luiz Eduardo Ceccon Calil:** Writing – original draft, Formal analysis, Conceptualization. **Bittante Carolina Devite:** Writing – review & editing, Formal analysis.

## Declaration of Competing Interest

The authors declare that there is no competing interest.

## Data Availability

All data can be found in Hospital Israelita Albert Einstein
